# Antibiotic resistance pattern of methicillin-resistant *Staphylococcus aureus* and *Escherichia coli* from mobile phones of healthcare workers in public hospitals in Ghana

**DOI:** 10.11604/pamj.2022.41.259.29281

**Published:** 2022-03-30

**Authors:** Courage Kosi Setsoafia Saba, Francis Naa-Inour, Stephen Wilson Kpordze

**Affiliations:** 1 Department of Biotechnology, Faculty of Agriculture, University for Development Studies, Tamale, Ghana,; 2 Department of Ecotourism and Environmental Management, Faculty of Natural Resources and Environment, University for Development Studies, Tamale, Ghana

**Keywords:** Healthcare workers, mobile phones, *Escherichia coli*, hospital acquired infections, antibiotic resistance

## Abstract

**Introduction:**

mobile phone plays an essential role in the lives of healthcare professionals in hospitals as far as communication is concerned. However, it can also serve as a source of nosocomial infections. This study aimed at determining the prevalence and antibiotic susceptibility of Methicillin-resistant Staphylococcus aureus (MRSA) and Escherichia coli isolated from mobile phones used by healthcare staff working in three public hospitals in Ghana.

**Methods:**

in total, 220 swab samples were collected from 110 mobile phones of healthcare workers at a referral and two public tertiary hospitals in Ghana. Direct spreading of swab samples on agar plates was done. MacConkey agar and Baird Parker agar were used to isolate E. coli and S. aureus, respectively. Clinical Laboratory Standard Institute´s guidelines were followed for susceptibility testing, and S. aureus strains resistant to cefoxitin were considered to be MRSA. All E. coli and MRSA isolates were tested for their susceptibility to antibiotics using European Committee on Antimicrobial Susceptibility Testing (EUCAST) 2018 guidelines with its breakpoints. Obtained qualitative data were analyzed by using Microsoft Excel.

**Results:**

of 110 mobile phones, 78 (70.9%) and 4 (3.6%) were colonized with S. aureus and E. coli, respectively. From the 78 S. aureus isolates, 22 (28%) isolates were MRSA. Fifty percent (50%) (11/22) of the MRSA isolates were multi-drug resistant, of which one isolate was resistant to all antibiotics tested. E. coli isolates had 100 resistances to both ceftriaxone and ceftazidime.

**Conclusion:**

mobile phones used by healthcare workers in hospitals frequently harbor E. coli, S. aureus, MRSA and may be sources of hospital-associated infections.

## Introduction

*Staphylococcus aureus* and *E. coli* along with their other species are both communal bacteria and opportunistic pathogens and their infections are common in low and middle income countries [1]. Approximately 30% of the human population is colonized with *S. aureus* [2] and the risk of developing this infection is increased in healthcare settings. In an estimate of every 100 hospitalized patients at any given time, seven in high-income and ten in low and middle income countries will acquire at least one hospital infection [3]. Methicillin-resistant *S. aureus* (MRSA) has emerged as a virulent pathogen and a leading cause of nosocomial infections [4], accounting for 20% to 80% of nosocomial infections [4]. Of current clinical concern, is the prevalence of multi-drug resistant MRSA in many parts of the world. In the United State (US), more than 40% of *S. aureus* blood stream infections are caused by MRSA [5]. In Europe, it ranges widely and the rates in the Mediterranean and United Kingdom (UK) exceed 30%, while the Netherlands and Scandinavian countries are approximately 2% [6]. Sources of these infections in hospitals include medical staff or colonized healthcare workers, colonized patients or patients´ own flora, contaminated environmental surfaces, and inanimate hospital objects [7]. Healthcare workers are increasingly exposed to colonized patients and, hence, more likely to be colonized with such infections than any ordinary person in the general population [8,9].

Several studies on clinicians´ mobile phones contamination in the USA and UK reported a level of overall mobile phone contamination (pathogenic and non-pathogenic organisms) ranging from 75% to 96%, with between 9% and 25% Methicillin-resistant *S. aureus* (MSSA), MRSA, *E. coli* [10,11]. *E. coli*, MRSA, and other virulent pathogens have been isolated from clinicians´ mobile phones; 98.6% MRSA has been detected from phones, hands, and ears of clinicians [11,12]. *E. coli* has been widely used as an indicator of fecal contamination instead of total coliform, which is more general and may include other environmental isolates. Therefore, *E. coli* was isolated as an indicator organism in this work to check if healthcare workers were practicing good hand hygiene. Little or no research has been conducted to assess bacteria on health workers´ mobile phones in Ghana. A previous study conducted by Saba *et al*. [13] assessed pathogens on door handles and other points of contact at the same hospitals considered in this study. The extent of knowledge about the potential of mobile phones to transmit infections among healthcare workers in Ghana was not known. This study, therefore, aimed at determining the antibiotic resistant profiles of *S. aureus*, Methicillin-resistant *S. aureus* (MRSA) and of *E. coli* isolates from mobile phones used by healthcare workers in three public hospitals in Ghana.

## Methods

**Study design:** the study was carried out at the three major government hospitals in the Tamale Metropolis of Ghana: i) Tamale Teaching Hospital (TTH): a referral hospital for three regions of Ghana, with 478 staff; ii) Tamale Central Hospital (TCH): a public tertiary healthcare facility with 156 beds and 442 staff; iii) Tamale West Hospital (TWH): a public tertiary healthcare center with 126 beds and 370 staff.

**Sample size:** samples were collected in March and April 2016. Permissions were granted by all three hospitals to conduct this research. Two hundred and twenty (220) swab (MEUS, Italy) samples were taken: 110 swabs for the isolation of S. aureus and 110 swabs for the isolation of *E. coli*. Samples were collected from the mobile phones of 110 healthcare workers. Out of the total 220 swab samples, 100 (50 mobile phones) were taken from the TTH, 60 (30 mobile phones) from TCH and 60 (30 mobile phones) from TWH. Mobile phones of health personnel at the administrative units were also swabbed as a control, since they do not have direct contact with patients. Individual sterile swabs moistened with sterile phosphate buffer saline (Oxoid, Hampshire, UK) were used to swab half the front and half the back surface of each mobile phone for *E. coli* isolation and the other half swabbed for *S. aureus* with a different swab. Two separate sterile swabs were used for each mobile phone, one for the isolation of *S. aureus* and the other for *E. coli* All samples were stored below 4°C and transported to the laboratory for analysis within 2 hours. All swab samples were then directly streaked onto MacConkey agar (Oxoid, Hampshire, UK) and Baird Parker agar (BioMerieux S.A, Spain), respectively for of *E. coli* and S. aureus. They were incubated for 24 hours at temperatures of 44.5°C and 37°C for E. coli and S. aureus, respectively. Suspected of *E. coli* colonies were further confirmed on Simmons citrate agar (Oxoid, Hampshire, UK) and incubated at 37°C for 24 hours. Pure cultures of E. coli and S. aureus were made by plating positive colonies onto nutrient agar (Oxoid, Hampshire, UK), prior to the susceptibility testing of both organisms. Antibiotic susceptibility testing was performed and interpreted according to the Clinical and Laboratory Institute Standard (CLSI) guidelines [14] to determine the cefoxitin resistant *S. aureus*. The breakpoints were determined using National Committee for Clinical Laboratory Standards (NCCLS) document M31-A2 [15]. Methicillin-Resistant *S. aureus* strains were classified as those isolate resistant to cefoxitin (30 µg) (BioMerieux, Spain). Similarly, antibiotic susceptibility tests were also performed on the positive of *E. coli* and the MRSA isolates using the EUCAST, 2018 guidelines with its breakpoints. The antibiotics disks used for the *E. coli* isolates were ciprofloxacin (5 µg), gentamicin (10 µg), erythromycin (15 µg), ceftriaxone (30 µg), ceftazidime (10 µg) and nitrofurantoin (100 µg). For the susceptibility test of the MRSA isolates, the antibiotic disks used were ciprofloxacin (5 µg), gentamicin (10 µg), erythromycin (15 µg), tetracycline (30 µg), and chloramphenicol (30 µg). Multi-drug resistant isolates were classified as resistance to three or more antibiotics. All isolates have been stored in 25% glycerol at -20°C, and are available for future studies.

**Statistical analysis:** data obtained were analyzed using Microsoft Excel (Microsoft Office 2016). Results were enumerated in frequencies and percentages and presented in tables and charts.

## Results

**Sources of *S. aureus* and *E. coli*:** a total of 78 S. aureus isolates were cultured from 78 (70.9%) phones of the 110 mobiles phones sampled. Of the 78 *S. aureus* 58% (29/50) were from TTH, 80% (24/30) from TWH and 83% (25/30) from TCH (Table 1). Among the 110 samples collected for the isolation of *E. coli* from the 110 different mobile phones, there were no mobile phones contaminated at TWH and TCH, but 8% (4/50) of mobile phones from TTH were contaminated with *E. coli*. The phones contaminated with *E. coli* were found on mobiles phones of personnel from the following wards: administration, children´s, medical, and male wards. All mobile phones contaminated with *E. coli* were also contaminated with *S. aureus*

**Table 1:** prevalence of *S. aureus* on mobile phones at the various departments/wards at each hospital

Department/hospital	TTH (%)	TCH (%)	TWH (%)	Total
Administration	5/11 (45.5%)	5/5 (100%)	5/5 (100%)	15/21 (71%)
OPD	2/4 (50%)	2/3 (67%)	3/3 (100%)	7/10 (70%)
Children ward	4/5 (80%)	2/4 (50%)	4/4 (100%)	10/13 (77%)
Female ward	4/6 (67%)	5/5 (100%)	1/4 (25%)	10/15 (67%)
Male ward	3/6 (50%)	3/4 (75%)	2/4 (50%)	8/14 (57%)
Surgical ward	4/5 (80%)	-	4/4 (100%)	8/9 (89%)
Maternity ward	4/5 (80%)	3/4 (75%)	3/4 (75%)	10/13 (77%)
Laboratory	2/5 (40%)	-	-	2/5 (40%)
Medical ward	1/3 (33%)	-	-	1/3 (33%)
Labour ward	-	3/3 (100%)	-	3/3 (100%)
Theatre	-	2/2 (100%)	2/2 (100%)	4/4 (100%)
Total	29/50 (58%)	25/30 (83%)	24/30 (80%)	78/110 (70.9%)

TTH: Tamale Teaching hospital; TCH: Tamale Central hospital; TWH: Tamale West hospital; OPD: out-patient department

**Determination of methicillin-resistant (MSRA) and methicillin-sensitive (MSSA) isolates using cefoxitin:** antibiotic susceptibility tests were performed on the 78 positive S. aureus samples. Of the total 78 positive S. aureus isolates, 28% (22) isolates were resistant to cefoxitin while 72% (56) were susceptible. Twenty-eight percent (28%) of the isolates were, therefore, considered as MSRA while 72% were classified as MSSA. Of the 28% MRSA isolates, 45% (10), 41% (9), 14% (3) were from TTH, TCH and TWH, respectively (Table 2). Table 3 shows the wards in the various hospitals where MRSA were isolated.

**Table 2:** methicillin-resistant *Staphylococcus aureus* and MSSA prevalence on mobile phones in the various hospitals

MRSA and MSSA Prevalence			
Susceptibility	Strain type	Number of isolates (%)	
Resistant to cefoxitin	MRSA	22 (28)	
Susceptible to cefoxitin	MSSA	56 (72)	
Total	*S. aureus*	78 (100)	
**Hospitals**	***S. aureus***	**MRSA Prevalence**	**MSSA Prevalence**
		**Freq (%)**	**Freq (%)**
TTH	29	10 (45)	19 (34)
TCH	25	9 (41)	16 (28.5)
TWH	24	3 (14)	21 (37.5)
Total	78	22 (100)	56 (100)

TTH: Tamale Teaching hospital; TCH: Tamale Central hospital; TWH: Tamale West hospital; MRSA: methicillin resistant Staphylococcus aureus; MSSA: methicillin susceptible *Staphylococcus aureus*; Freq: frequency; values in bracket indicate percentage

**Table 3:** wards/departments of personnel at the time samples were taken

Sample code	Hospital	Ward/department
4TTH	TTH	Administration
8TTH	TTH	Administration
13TTH	TTH	OPD
17TTH	TTH	Medical ward
20TTH	TTH	Children's ward
21TTH	TTH	Children's ward
32TTH	TTH	Female ward
39TTH	TTH	Female ward
46TTH	TTH	Maternity ward
50TTH	TTH	Male ward
9TWH	TWH	Children's ward
21TWH	TWH	Theatre
31TWH	TWH	Labour ward
2TCH	TCH	Administration
6TCH	TCH	OPD
9TCH	TCH	OPD
13TCH	TCH	Female ward
14TCH	TCH	Maternity ward
19TCH	TCH	Male ward
22TCH	TCH	Labour ward
27TCH	TCH	Theatre
30TCH	TCH	Children's ward

TTH: Tamale Teaching Hospital; TCH: Tamale Central Hospital; TWH: Tamale West Hospital; OPD: out-patient department

**Antibiotic resistance profile of MRSA isolates:** fifty percent (11/22) of the MRSA isolates from the mobile phones were multi-drug resistant MRSA. One MRSA isolate, which was taken from a mobile phone on the female ward of the TCH, was resistant to all the five antibiotics (ciprofloxacin, gentamicin, erythromycin, tetracycline, and chloramphenicol) while another isolate, which was taken from the children´s ward of TCH, was resistant to four (gentamicin, erythromycin, tetracycline, and chloramphenicol) out of the five antibiotics tested (Table 4). Nine (9) of the 22 isolates were resistant to three antibiotics of which four were from the TTH (administration block, children´s, female and maternity wards), four from the TCH (administration block, OPD, male and labor wards) and one was from TWH (children´s ward) (Table 4). Six (27.3%) of the isolates were resistant to two antibiotics and three (13.6%) isolates were resistant to only one antibiotic. Twenty (90.9%) of the isolates were resistant to one or more antibiotics, while two isolates were susceptible to all the antibiotics used. Only one MRSA isolate (4.5%) was resistant to ciprofloxacin, two (9%) isolates were resistant to gentamicin, fourteen (63.6%) isolates were resistant to tetracycline, sixteen (72.72%) isolates were to erythromycin, and eighteen (81.82%) isolates were resistant to chloramphenicol. The highest resistance was recorded against chloramphenicol and the least was ciprofloxacin (Table 4). In Table 4 below shows the resistant profile of the MRSA and various hospital sources.

**Table 4:** antibiotic resistant profile of MRSA and their sources

Antibiotics	MRSA Susceptibility Profile (Freq/%)		
	R	I	S
CIP	1 (4.55)	0	21 (95.45)
GMN	2 (9.09)	0	20 (90.91)
ERY	16 (72.72)	3 (13.64)	3 (13.64)
TET	14 (63.64)	2 (9.09)	6 (27.27)
C	18 (81.82)	0	4 (18.18)
	**MRSA resistant profile of the various hospitals**		
**MRSA (n = 22)**	**Resistance profile**	**Isolates per hospital**	**Sources**
**Number of MRSA isolates**	**Number and list of antibiotics**		**Hospital/department or wards**
1 (4.5)	5 = CIP, GMN, ERY, TET, C	1 (TCH)	TCH = female ward
1 (4.5)	4 = GMN, ERY, TET, C	1 (TCH)	TCH = children's ward
9 (40.9)	3 = ERY, TET, C	4 (TTH)	TTH = administration block, children's, female and maternity ward
		4 (TCH)	TCH = administration block, OPD, male and labour wards
		1 (TWH)	TWH = children's ward
6 (27.3)	2 = TET, C or ERY, TET or ERY, C	2 (TTH)	TTH = administration and medical ward
		2 (TCH)	TCH = theatre and OPD
		2 (TWH)	TWH = theatre and labour ward
3 (13.6)	1 = ERY or C	2 (TTH)	TTH = OPD and female ward
		1 (TCH)	TCH = female ward
2 (9.1)	0	2 (TTH)	TTH = children's and male ward

TTH: Tamale Teaching hospital; TCH: Tamale Central hospital; TWH: Tamale West hospital; OPD: out-patient department, MRSA: methicillin-resistant *Staphylococcus aureus*; Freq: frequency; n: number; CIP; ciprofloxacin; GMN: gentamicin; ERY: erythromycin; TET: tetracycline; C: chloramphenicol; R: resistant; I: intermediate; S: susceptible (values in bracket indicate percentage)

**Antibiotic resistance pattern of the *E. coli* isolates:** antibiotic susceptibility tests were performed on the four (3.4%) positive *E. coli* isolates using six different antibiotics (ciprofloxacin (5µg), gentamicin (10µg), erythromycin (15µg), ceftriaxone (30µg), ceftazidime (10µg) and nitrofurantoin (100µg). None of the isolates was exhibited multi-drug resistance. The resistant pattern can be found in Table 5. All the four (100%) isolates were resistant to both ceftriaxone and ceftazidime.

**Table 5:** resistance pattern of isolated *E. coli*

Sample code	Antibiotics					
	CIP (5 µg)	GMN (10 µg)	ERY (15 µg)	CRO (30 µg)	CAZ (10 µg)	NIT (100 µg)
4TTH	S	S	S	R	R	S
21TTH	S	S	S	R	R	S
42TTH	S	S	S	R	R	S
50TTH	S	S	S	R	R	S

CIP= ciprofloxacin; GMN= gentamicin; ERY= erythromycin; CRO= ceftriaxone; CAZ= ceftazidime; NIT= nitrofurantoin; S= susceptible; R= resistant

## Discussion

Our results provide an essential informative baseline on *S. aureus, E. coli* and MRSA on mobile phones belonging to healthcare workers of these hospitals in Ghana. There have been previous reports on the possibility of pathogens transmissions, not only by mobile phones, but also other electronic devices such as personal digital assistants, handheld computers, stethoscopes, which also included some epidemiologically important drug-resistant pathogens [16]. High level of *S. aureus* colonization in this study could be as a result of improper hand hygiene practice and irregular sensitization of mobile phones by these health workers as they come into contacts with colonized patients, surfaces, objects, and other objects in the hospital environments. This is in accordance with Katsuse-Kanayama *et al*. [17] and reinforces the need for proper hand hygiene practice by healthcare workers prior to patient contact as a critical way of avoiding healthcare-associated infections. Though the rate of *E. coli* contamination was low in our study, it is higher than the rates (0.98%) reported by Morubagal *et al*. [18] but similar to Rana *et al*. [19], where they reported 2%. Conversely, Tagoe *et al*. [20], Selim and Abaza [21], and Debnath *et al*. [22] had little higher rates of 8%, 13% and 14%, respectively. Contamination with *E. coli* means that other fecal coliforms and pathogens from healthcare workers´ mobile phones could be transmitted onto their hands and subsequently to the patients they serve or other common contact surfaces in the hospitals.

The MRSA isolation rate found in this study (28%) is higher than similar researches carried out. Oguzkaya-Artan *et al*. [23] recorded 0.95% rate of isolation in Turkey, 1.4% by Heyba *et al*. [24] in Kuwait, 11.7% by Smibert *et al*. [25] in Australia, and 13% by Pal *et al*. [26] in the UK. However, other researchers reported higher isolation rates such as 53% by Selim and Abaza [21] in Egypt, 37.5% by Chang *et al*. [27] in Taiwan, 73% by Iyer *et al*. [9] in Saudi Arabia, 33.9% by Shibabaw *et al*. [8] in Ethiopia and 53.3% by Angadi *et al*. [28] in India. Fifty percent (11/22) of the MRSA isolates from the mobile phones were multi-drug resistant MRSA isolates (Table 4). It is worrisome to find that an isolate from one of the mobile phones was resistant to all the antibiotics tested. There is a risk of resistant microbes and genes being possibly transferred to healthcare workers or patients. This could pose an increased financial burden on patients with low income status. These patients may be forced to resort to other advanced line antibiotic treatments, which are more expensive. Other previous studies did record higher percentages of *E. coli* contamination on mobile phones than our work [12,29]. Per the phenotypic resistance pattern, they may be similar or the same strains, but further work needs to be done to ascertain it. The presence of faeces on mobiles phones may be due to lack of cleanliness and low hygiene standards [22].

The antibiotic resistance profile of our study showed no multi-drug resistance (i.e. resistance to three or more antibiotics), but all the *E. coli* isolates were 100% resistant to both ceftazidime and ceftriaxone unlike other studies where they recorded 42.9% resistance to ceftazidime [22] and 50% resistance to ceftriaxone [18]. Our study also recorded 100% susceptibility to ciprofloxacin, gentamicin, erythromycin and nitrofurantoin but Debnath *et al*. [22] reported 42.9% and 57.1% resistance to both gentamicin and ciprofloxacin respectively in Bangladesh, and Morubagal *et al*. [18] reported 50% resistance to ciprofloxacin in Iran. However, our study had similar results with Gashaw *et al*. [30] who found *E. coli* isolates from healthcare workers´ mobile phones to be 100% sensitive to ciprofloxacin and gentamicin. On two of the mobile phones, both E. coli and MRSA were isolated. One mobile phone had ceftriaxone and ceftazidime resistant *E. coli* and erythromycin, tetracycline and chloramphenicol resistant MRSA. The other mobile phone had *E. coli* resistant to ceftriaxone and ceftazidime, but the MRSA isolate from it was susceptible to all the antibiotics used. Further molecular characterization of these strains is required to understand the resistance dynamics, which was a limitation of this particular research.

**Limitations of the study:** it would have been relevant to extend the study to smaller and/or private hospitals. This study was limited to public hospitals at Tamale metropolis. Careful consideration should be taken to avoid generalizing the findings to all hospitals in Tamale metropolis. Data limitation on sample size was a major obstacle, especially from Ghana. The researchers used journals for information on Africa, and relied on the little available literature that was available for estimation. The study employed conventional approach instead of molecular (PCR) methods that are known to be highly specific. To overcome this, Baired Parker supplemented with Rabbit Plasma Fibrinogen (RPF) which makes it highly selective for *S. aureus* isolation, and use of biochemical confirmation were employed for the study.

## Conclusion

This study is the first to focus on contamination of healthcare workers´ mobile phones with *E. coli* and *S. aureus* in Ghana. In three hospitals in Tamale, health worker´s phones recorded very low colonization of *E. coli* but there was high prevalence with S. aureus exhibiting relatively high rates of MRSA that presented various resistance patterns to the antibiotic used in the study. Health workers´ mobile phones may serve as a source of transmission of MRSA to patients. Awareness creation on the potential for mobile phones to spread diseases in the hospital setting, alongside with intensive programs on infection prevention and control in hospitals, is recommended.

### What is known about this topic

- Presence of E. coli on fomites indicates fresh faecal contamination;
- Staphylococcus aureus is among the leading isolates in nosocomial infections;
- Methicillin-resistant S. aureus strains are resistance to multiple drugs.

### What this study adds

- Our study allowed us to have current data on the MRSA and E. coli prevalence on healthcare workers' mobile phones (rates of 28% MRSA and 0.98% E. coli) in three main public hospitals in the Northern region of Ghana;
- Some healthcare workers had their mobile phones contaminated with fresh faeces (presence of E. coli); however, these isolates were not multidrug resistant;
- This study discovered the high level of colonization of health workers' mobile phones with MRSA isolates, which are multi-drug resistant; these results will be very useful to the hospitals' infection prevention and control (IPC) departments to take significant measures to curb the spread of nosocomial diseases.
